# Enhancing Chaos Complexity of a Plasma Model through Power Input with Desirable Random Features

**DOI:** 10.3390/e23010048

**Published:** 2020-12-30

**Authors:** Hayder Natiq, Muhammad Rezal Kamel Ariffin, Muhammad Asyraf Asbullah, Zahari Mahad, Mohammed Najah

**Affiliations:** 1 Information Technology Collage, Imam Ja’afar Al-Sadiq University, Baghdad 10001, Iraq; hayder.natiq@sadiq.edu.iq; 2 Institute for Mathematical Research, Universiti Putra Malaysia, UPM, Serdang 43400, Malaysia; ma_asyraf@upm.edu.my (M.A.A.); zaharimahad@upm.edu.my (Z.M.); 3 Department of Mathematics, Faculty of Science, Universiti Putra Malaysia, UPM, Serdang 43400, Malaysia; 4 Centre of Foundation Studies for Agriculture Science, Universiti Putra Malaysia, UPM, Serdang 43400, Malaysia; 5 Institute of Informatics and Computing in Energy, Universiti Tenaga Nasional, Kajang 43000, Malaysia; najah.mahdi@uniten.edu.my

**Keywords:** transient chaotic behavior, multistability, sample entropy, randomness

## Abstract

The present work introduces an analysis framework to comprehend the dynamics of a 3D plasma model, which has been proposed to describe the pellet injection in tokamaks. The analysis of the system reveals the existence of a complex transition from transient chaos to steady periodic behavior. Additionally, without adding any kind of forcing term or controllers, we demonstrate that the system can be changed to become a multi-stable model by injecting more power input. In this regard, we observe that increasing the power input can fluctuate the numerical solution of the system from coexisting symmetric chaotic attractors to the coexistence of infinitely many quasi-periodic attractors. Besides that, complexity analyses based on Sample entropy are conducted, and they show that boosting power input spreads the trajectory to occupy a larger range in the phase space, thus enhancing the time series to be more complex and random. Therefore, our analysis could be important to further understand the dynamics of such models, and it can demonstrate the possibility of applying this system for generating pseudorandom sequences.

## 1. Introduction

Further investigations in nonlinear dynamical systems have contributed to the development and understanding of numerous scientific phenomena. In particular, chaotic models as nonlinear systems have been derived from rules for describing complex behaviors. For instance, the Lorenz model is the 3D chaotic system proposed to represent the atmospheric convection [1], and the logistic map is the 1D discrete-time chaotic model proposed to describe the population growth [2].

Although chaotic systems are deterministic, which indicates that the whole future path of the system is uniquely governed by its initial condition, it is impossible to predict its long-term behavior [3,4]. These interesting systems have attracted considerable interest, especially in the area of telecommunication, security, laser, engineering, and many others [5,6,7,8,9]. Moreover, chaotic behavior can be observed in many disciplines of science such as physics, chemistry, biology, economy, and nature [10,11,12,13]. Particularly, it has been reported several plasma models exhibiting chaos. Some examples are as follows. The authors of [14] discovered the developed and weak chaotic motions in a magnetized dusty plasma model. The authors of [15] studied a new Lorenz-like system describing the interaction of three resonantly coupled waves in plasma. The authors of [16] presented the mechanical analysis and proposed a supremum bound of a plasma chaotic attractor. The authors of [17] interpreted the dynamics of a mechanism plasma chaotic system. The authors of [18] proposed a method for finding hidden chaotic attractors in the plasma chaotic system.

The analysis of equilibrium points of chaotic systems has contributed to better understand their dynamics [19,20] and for determining their attractor type [21]. In general, there are two major types of chaotic attractors: the first type is called self-excited, and the second type is called hidden. The self-excited attractors can be found in chaotic systems that have a basin of attraction intersected with an unstable equilibrium [22]. Meanwhile, hidden attractors can be found in systems with a line of equilibria [23], curves of equilibria [24], no equilibria [25], or infinite number of equilibria [26].

The investigations of hidden and self-excited chaotic attractors have revealed unexpected results known as coexisting attractors or multistability, which has given the chaotic systems another dimension [27,28]. A gas laser model is the first system that shows multistability [29]; since that time many models with coexisting attractors have been presented [30,31,32,33,34]. From the application point of view, multistability provides large flexibility in the performance of nonlinear dynamical systems without changing system parameters [35,36]. Therefore, it can be used to induce switching between desirable attractors [27]. However, the multistability behavior can be harmful, especially in the design of a commercial device with particular properties where it is substantial to stabilize the required state in the existence of noise [37]. Additionally, the fluctuation of a chaotic system from chaos to the periodic state could break the chaos-based cryptosystems [30].

In this paper, we investigate the dynamics of a plasma perturbation, which consists of three coupled ordinary differential equations that contain three parameters [38]. The stability of equilibria reveals that the emergence of a chaotic attractor by the system is self-excited. Moreover, complex nonlinear behavior of transition from transient chaos to steady periodic can be observed by times series and phase space. To show the chaotic regions, which show no transient chaos, we use maximum Lyapunov exponents based on the contour plot. However, the system shows other complex behaviors of coexisting multiple symmetric attractors, which can be generated without adding forcing terms or controllers by increasing the power input. On this matter, boosting power input not only generates extreme multistability, but also can enhance the complexity and randomness of the system time series.

The remainder of this paper is organized as follows. Section 2 investigates the stability of the equilibria of the plasma perturbation model and determines its chaotic regions. Section 3 shows the ability of the plasma system for generating various multistability behaviors by increasing its power input. Section 4 illustrates the effect of increasing power input on the complexity of the system. In Section 5, we demonstrate the randomness of the system and show its high sensitivity to the initial conditions. Section 6 provides the conclusion of the paper.

## 2. The Mathematical Model

This paper concentrates on a low-dimensional model [38], that describes the dynamics of pellet injection in tokamaks. Mathematically, it is given by

(1) $$\begin{array}{c} \left\{\begin{array}{c} \begin{array}{cc} \frac{d^{2}}{dt_{n}^{2}}\xi_{n}= & \left(p_{n}^{\prime }-1\right)\cdot \xi_{n}-\delta \cdot \frac{d}{dt_{n}}\xi_{n},\\ \frac{d}{dt_{n}}p_{n}^{\prime }= & \eta \cdot (h-p_{n}^{\prime }-\frac{\chi_{anom}}{\chi_{0}}\cdot \xi_{n}^{2}\cdot p_{n}^{\prime }), \end{array} \end{array}\right. \end{array}$$

where $p_{n}^{\prime }$ is the normalized plasma pressure gradient, $\xi_{n}$ is the normalized magnetic field, and χ is the thermal diffusivity.

However, the corresponding 3D dynamical model can be found as follows,

(2) $$\begin{array}{c} \left\{\begin{array}{c} \begin{array}{cc} \frac{dx_{1}}{dt} & =x_{2}x_{3}-\left(x_{2}+dx_{1}\right),\\ \frac{dx_{2}}{dt} & =x_{1},\\ \frac{dx_{3}}{dt} & =eh-e\left(x_{2}^{2}+1\right)x_{3}, \end{array} \end{array}\right. \end{array}$$

which was obtained by introducing the variables $x_{2}=\sqrt{\frac{\chi_{anom}}{\chi_{0}}}\cdot \xi_{n}$ and $x_{3}=p_{n}^{\prime }$. The constant parameters in the system (2) are defined as follows: *d* represents the dissipation/relaxation of perturbation, *e* represents the characteristic relation between the two heat diffusion coefficients, and *h* is the power input into the system.

We now give the basic features of the system (2) to interpret its complex behavior. First, the system (2) is symmetric about $x_{3}-$axis, and this is due to that it is remained unaltered under the transformation $\left(x_{1},x_{2},x_{3}\right)⟶\left(-x_{1},-x_{2},x_{3}\right)$. Second, it is dissipative only when the state space contraction, which is defined by $\left(d+e+ex_{2}^{2}\right)$, is greater than zero. Finally, three different equilibrium points—$P_{1}\left(0,\;0,\;h\right)$, $P_{2}\left(0,\;\sqrt{h-1},\;1\right)$, and $P_{3}\left(0,-\;\sqrt{h-1},\;1\right)$—can be obtained by considering the left hand sides of the three equations in the system (2) equal to zero.

For studying the stability of the system (2) at the equilibrium point $P_{1}$, we first obtain the corresponding eigenvalues, which are given by

(3) $$\begin{array}{c} \lambda_{1}=-e,\;\lambda_{2,3}=\frac{-d}{2}\pm \frac{1}{2}\sqrt{d^{2}+4h-4}. \end{array}$$

A continuous dynamical system is unstable when its eigenvalues have positive real parts. Obviously, $\lambda_{1}$ is positive only when the parameter *e* is less than zero. Meanwhile, $\lambda_{2,3}$ have positive real parts if h>1. Moreover, the characteristic equation at $P_{2,3}$ is given by

(4) $$\begin{array}{c} \lambda^{3}+\left(d+eh\right)\lambda^{2}+deh\lambda +2e\left(h-1\right)=0. \end{array}$$

Suppose that the parameters d,e,h are greater than zero. Then, the eigenvalues at $P_{2,3}$ have negative real parts when

(5) $$\begin{array}{cc} & d+eh>0, \end{array}$$

(6) $$\begin{array}{cc} & 2eh-2e>0, \end{array}$$

(7) $$\begin{array}{cc} & d^{2}h+edh^{2}>2\left(h-1\right). \end{array}$$

Thus, the equilibrium points $P_{2,3}$ are unstable when one of the above inequalities does not hold. In other words, the system (2) at $P_{2,3}$ is unstable if and only if

(8) $$\begin{array}{c} \frac{2(h-1)}{dh\cdot (eh+d)}>1. \end{array}$$

### 2.1. Self-Excited Chaotic Attractor and Complex Transient Chaos

**Figure 1:**
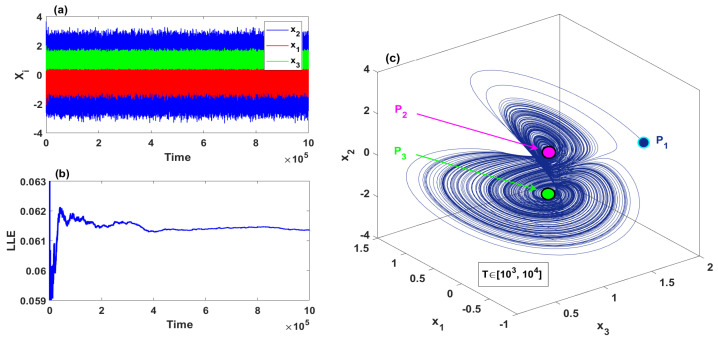
Self-excited chaotic attractor with d=0.5, e=0.1, h=2 and for the initial conditions (0.0001,0.0001,h): (**a**) the time series; (**b**) largest Lyapunov Exponents; (**c**) the phase space.

When the parameters values set as d=0.5, e=0.1, h=2, Figure 1a–c depicts the time series, largest Lyapunov Exponents, and the phase space of the system (2), respectively. For the numerical simulation of the system (2), we use Matlab 2019a program to plot the time series, the phase spaces, and largest Lyapunov Exponents, in which the standard Runge–Kutta fourth-order scheme was utilized via Matlab to obtain the system solution and then plot the time series and the phase spaces, whereas Wolf algorithm [39] was utilized to calculate the largest Lyapunov Exponent in Matlab. However, it can be seen that the system exhibits chaotic attractor for a long duration time when the starting point is from a neighborhood of the equilibrium point $P_{1}$. As all equilibria of the system (2), including $P_{1}$, are unstable for d=0.5, e=0.1, h=2, the generated chaotic attractor in Figure 1 is self-excited.

**Figure 2:**
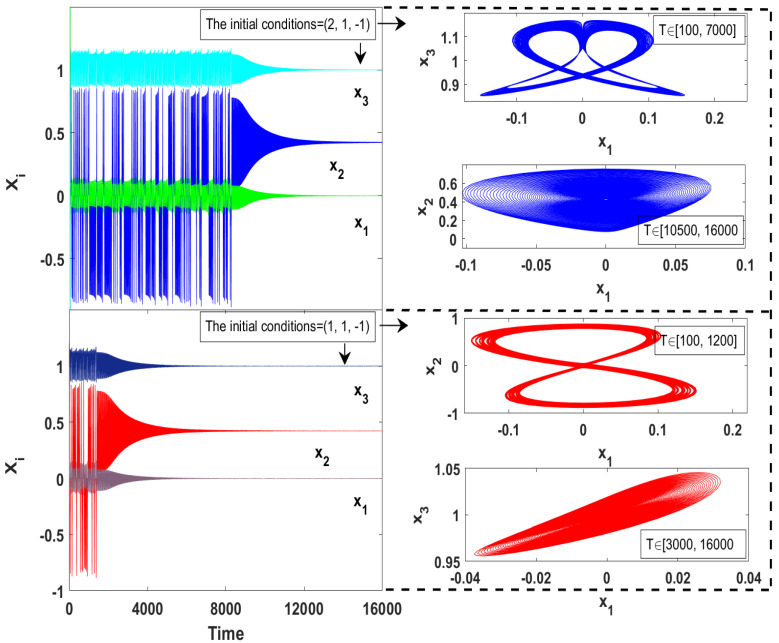
Chaotic transient of the system (2) with d=0.5, e=0.1, h=1.179 and for two different sets of initial conditions.

On the other hand, transient chaos usually appears when the initial conditions are chosen near the boundary of the basin of attraction or when the system is very close to a bifurcation point. Thus, by choosing the parameters values as d=0.5, e=0.1, h=1.179, which make the system is close enough to a bifurcation point, we observe that the system (2) exhibits a complex chaotic transient, as shown in Figure 2. In this figure, we have plotted the time series and the corresponding phase portraits for a specific set of the system parameters and with two different sets of initial conditions, which have been chosen near the boundary of the basin of attraction. As can be seen, for each set of the initial conditions, all variables of the system ($x_{1},x_{2},x_{3}$) show a chaotic behavior in short duration time, and subsequently those variables show asymptotic orbit for the rest of the time.

### 2.2. Chaotic Regions of the Plasma Model

**Figure 3:**
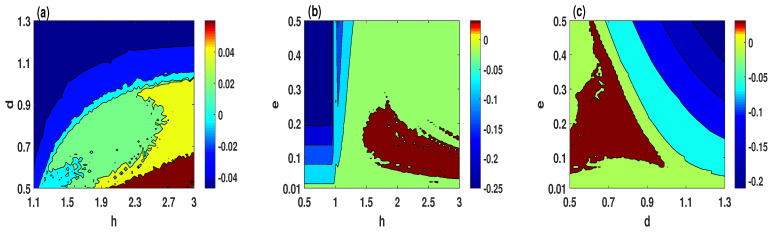
The largest Lyapunov exponent of the system (2) for the initial conditions (2,1,−1): (**a**) e=0.1; (**b**) d=0.5; (**c**) h=2.3.

Chaos is usually identified by one of the fundamental algorithms, which is Lyapunov exponent. The Lyapunov exponent is a quantitative measure employed to estimate the divergence or convergence of adjacent trajectories in a system. Here, we calculate the largest Lyapunov exponent when two parameters are varying simultaneously, which can provide a wider vision of the chaotic regions of the system. Therefore, three different sets of system parameters are selected to depict the largest Lyapunov exponent with a step size of 0.02, as shown in Figure 3. Based on this figure, it can be observed that the system (2) exhibits three different behaviors, which can be determined as follows.

- Figure 3a illustrates that the chaotic behavior appears in the brown, yellow, and green colors regions. Meanwhile, the quasi-periodic and periodic behaviors appear in cyan and blue colors regions, respectively.
- Figure 3b,c shows that the chaotic behavior of the system (2) appears in the brown color region, and the quasi-periodic behavior appears in the green color region. Meanwhile, the cyan and blue colors regions indicate to the periodic behavior of the system.

## 3. The Plasma Model with Coexisting Symmetric Attractors

In this section, we study the dynamical behaviors of the plasma model (2) by injection more power input to the system. This can be done by adding a constant parameter *A* to the third equation and fixing the diffusion parameters *e*. As a result, the modified system is defined as follows,

(9) $$\begin{array}{c} \left\{\begin{array}{c} \begin{array}{cc} \frac{dx_{1}}{dt} & =x_{2}x_{3}-\left(x_{2}+dx_{1}\right),\\ \frac{dx_{2}}{dt} & =x_{1},\\ \frac{dx_{3}}{dt} & =\left(eh+A\right)-e\left(x_{2}^{2}x_{3}+x_{3}\right), \end{array} \end{array}\right. \end{array}$$

where *A* is a positive parameter. Obviously, adding *A* to the third equation of the system (2) can keep the symmetry of the system about $x_{3}-$axis intact. Moreover, the system (9) has the same number of equilibria as in the system (2). These equilibria are given by

$$\begin{array}{c} P_{1}^{{}^{\prime }}\left(0,\;0,\;\frac{eh+A}{e}\right),\\ P_{2}^{{}^{\prime }}\left(0,\;\sqrt{\frac{eh+A}{e}-1},\;1\right),\\ P_{3}^{{}^{\prime }}\left(0,\;-\sqrt{\frac{eh+A}{e}-1},\;1\right). \end{array}$$

The stability of $P_{1}^{{}^{\prime }},P_{2}^{{}^{\prime }},P_{3}^{{}^{\prime }}$ is determined by the following proposition.

**Proposition** **1.** *The following statements hold if the parameters d,h,e,A are greater than zero.*

*(1)*  *$P_{1}^{{}^{\prime }}$ is saddle point if $\frac{eh+A}{e}>1$.*
*(2)*  *$P_{2,3}^{{}^{\prime }}$ are unstable equilibria if $\frac{A+e(h-1)}{d(A+eh+d)\cdot (A+eh)}>\frac{1}{2}$.*

### 3.1. The Coexistence of a Symmetric Pair of Attractors

A chaotic system, which exhibits different attractors for a particular set of system parameters, is considered as highly sensitive to its initial conditions. Therefore, this section discover the presence of coexisting attractors after the injection of more power input to the system.

**Figure 4:**
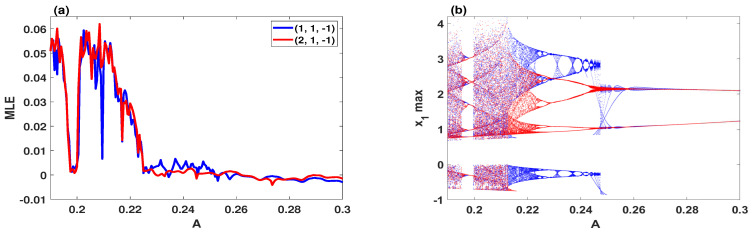
Dynamics of the system (9) with d=0.5, e=0.1, h=2.3 and two sets of initial conditions (1,1,−1) (blue) and (2,1,−1) (red): (**a**) the largest Lyapunov Exponent; (**b**) the coexisting bifurcation model.

By selecting *A* as control parameter for the range 0.19≤A≤0.3, the largest Lyapunov exponents and multistable bifurcation diagram of the system (9) are, respectively, plotted in Figure 4a,b when the step size is equal to 0.0001. By observing such the two figures, the system is simulated with two different initial conditions simultaneously, in which the blue and red orbits begin with the initial conditions (1,1,−1) and (2,1,−1), respectively. Obviously, the system exhibits single chaotic attractors when A∈[0.19,0.196]∪(0.2,0.2135), and single quasi-periodic attractors when A∈(0.196,0.2]∪(0.24,0.3]. Meanwhile, the coexistence of two chaotic attractors has appeared when A∈[0.2135,0.222], and the coexistence of two quasi-periodic attractors has occurred when A∈(0.222,0.24].

**Figure 5:**
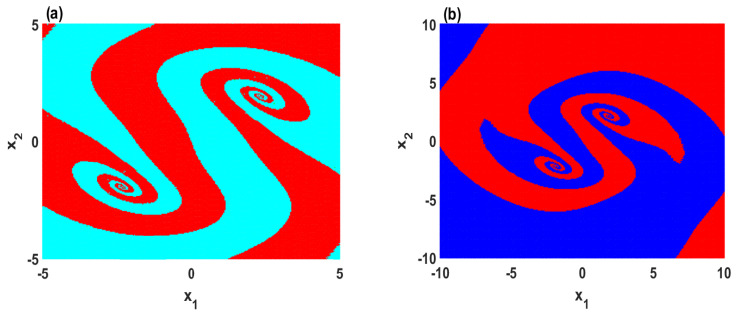
Cross section for z=−1 of the basins of attraction on $x_{1}$–$x_{2}$ plane with d=0.5,e=0.1,h=2.3: (**a**) the coexistence of two chaotic attractors for A=0.22; (**b**) the coexistence of two quasi-periodic attractors for A=0.24.

**Figure 6:**
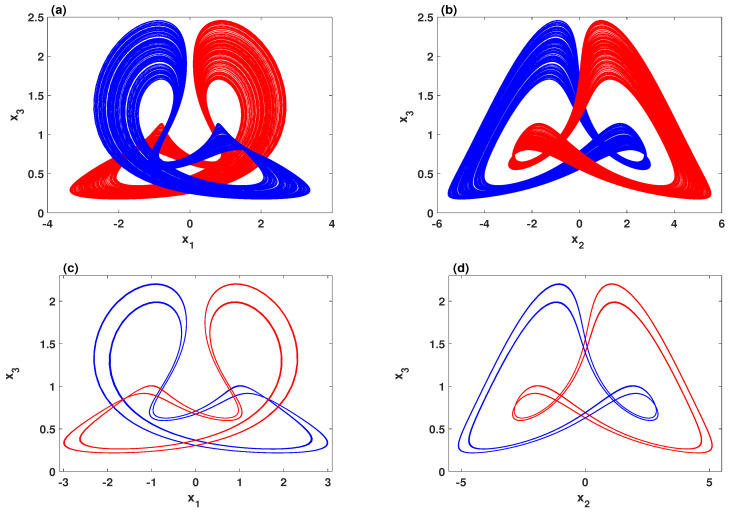
Symmetric coexisting attractors with d=0.5, e=0.1, h=2.3 and two sets of initial conditions (1,1,−1) (blue) and (2,1,−1) (red): (**a**,**b**) the coexistence of two chaotic attractors when A=0.22; (**c**,**d**) the coexistence of two periodic attractors for A=0.24.

It is crucial here to plot the basins of attraction to provide foresight about the behavior of the system with a wide range of initial conditions. Therefore, the basins of attraction of the system in two different scenarios are plotted: the parameters region where two chaotic attractors coexist, and the region of the parameter where two quasi-periodic attractors coexist, as illustrated in Figure 5a,b, respectively. For both scenarios, the basins of attractions agree with the bifurcation diagram in which the system exhibits two symmetric coexisting attractors. This can be further illustrated by depicting the corresponding phase portraits, as shown in Figure 6. It can be seen that the system with A=0.22 shows the coexistence of symmetric wings of strange attractors in which these attractors have formed a shape that resembles a butterfly, as shown in Figure 6a,b. Besides that, the coexistence of symmetric wings of double-period periodic attractors can be observed with A=0.24, as shown in Figure 6c,d. However, Table 1 lists the Kaplan–Yorke dimension (DKY) and Lyapunov exponents (LE) of the attractors that are shown in Figure 6. Here, we use the Wolf algorithm [39] to calculate the Lyapunov exponents with $t=10^{4}$.

### 3.2. The Coexistence of Many Symmetric Quasi-Periodic Attractors

**Figure 7:**
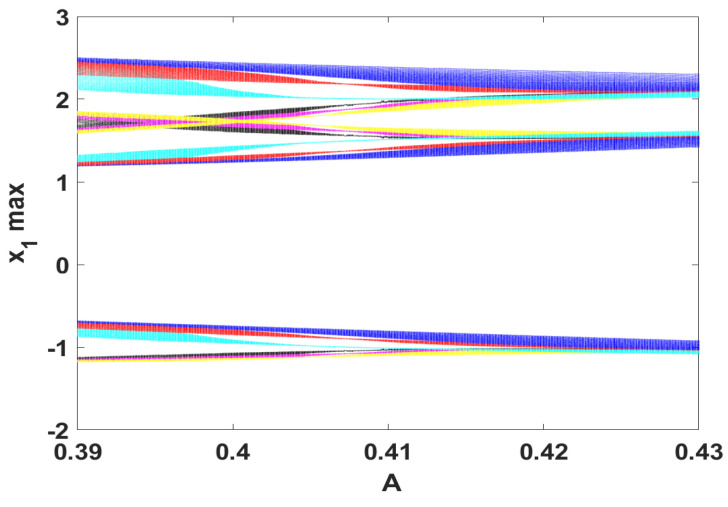
Coexisting bifurcation model with the parameters d=0.5, e=0.1, h=1.95 and for the initial conditions (K,1,−1), where *K* equals to −6 (cyan), −3 (green), −1 (blue), 0 (red), 2 (yellow), 4 (black), 5 (purple).

**Figure 8:**
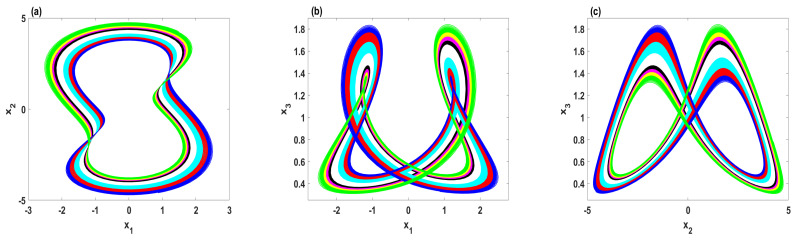
The phase portraits for the parameters e=0.1, d=0.5, h=1.95, a=0.4 and with (K,1,−1), where *K* equals to −6 (cyan), −3 (green), −1 (blue), 0 (red), 2 (yellow), 4 (black), 5 (purple): (**a**) $x_{1}$–$x_{2}$ plane; (**b**) $x_{1}$–$x_{3}$ plane; (**c**) $x_{2}$–$x_{3}$ plane.

To examine the effect of increasing the power input for the system, Figure 7 depicts the coexisting bifurcation diagram with respect to parameter *A*. In this figure, we set the parameters d=0.5, e=0.1, h=1.95 and 0.39≤A≤0.43. Meanwhile, the system is simulated with several sets of initial values, which are selected as (K,1,−1). When *K* is set to −6,−3,−1,0,2,4,5, the coexistence of seven quasi-periodic attractors is observed mainly within the range A∈[0.39,0.41]. This interesting nonlinear phenomenon can be further illustrated by plotting different projections of the system phase portraits, as shown in Figure 8.

## 4. Sample Entropy Algorithm

This section studies the complexity performance of systems (2) and (9) by one of fundamental algorithms, which is Sample Entropy (SamEn) [40]. SamEn is derived from approximate entropy by Richman et al. to estimate how much extra information is required to predict the (t+1)th output of a trajectory using its previous (t) outputs. Larger SamEn values reflect a lower degree of regularity, which means that higher complexity performance.

The SamEn algorithm for a given time series {x(i),i=0,1,2,…,N−1} is outlined as follows.

1. Reconstruction: the time series can be reconstructed as follows
  (10) $$\begin{array}{c} X_{i}=\left\{x_{i},x_{i+\tau },\ldots ,x_{i+(m-1)\tau }\right\},\;X_{i}\in R^{m} \end{array}$$
  where *m* is embedding dimension, and τ is time delay.
2. Counting the vector pairs: For a given tolerance parameter *r*, let $B_{i}$ be the number of vectors $X_{j}$ such that
  (11) $$\begin{array}{c} d[X_{i},X_{j}]\leq r,\;i\neq j \end{array}$$
  here, $d[X_{i},X_{j}]$ is the distance between $X_{i}$ and $X_{j}$, which is defined as
  (12) $$\begin{array}{cc} d\left[X_{i},X_{j}\right]=\max\left\{\right|x\left(i+k\right)- & x(j+k)|:\\ & 0\leq k\leq m-1\}. \end{array}$$
3. Calculating $\theta^{m}\left(r\right)$: According to the obtained number of vector pairs, we can get
  (13) $$\begin{array}{cc} C_{i}^{m}\left(r\right) & =\frac{B_{i}}{N-(m-1)\tau }, \end{array}$$
  then calculate $\theta^{m}\left(r\right)$ by
  (14) $$\begin{array}{cc} \theta^{m}\left(r\right) & =\frac{\sum_{i=1}^{N-(m-1)\tau }lnC_{i}^{m}\left(r\right)}{\left[N-(m-1)\tau \right]}. \end{array}$$
4. Calculating SamEn: Repeating the above steps we can get $\theta^{m+1}\left(r\right)$, then SamEn is given by
  (15) $$\begin{array}{c} SamEn\left(m,r,N\right)=\theta^{m}\left(r\right)-\theta^{m+1}\left(r\right). \end{array}$$

**Figure 9:**
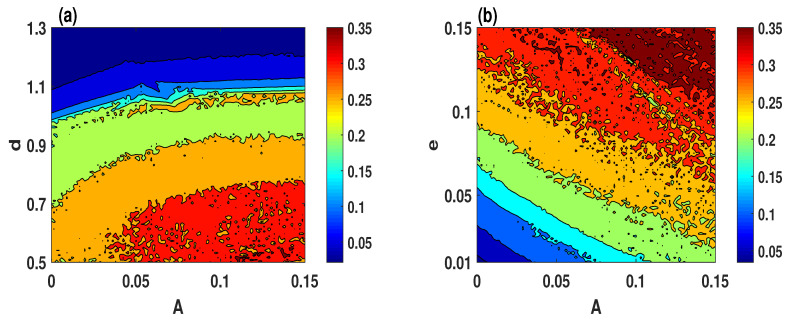
Complexity results of the system (9) with the initial conditions (1,1,−1): (**a**) e=0.1 and h=2; (**b**) d=0.55 and h=2.

For m=2 and r=0.2× Standard Deviation, we depict the complexity analysis results of the system (9) using Matlab 2019a program with two different sets of the system parameters, as shown in Figure 9a,b. There are two parameters simultaneously varying with the step size equal to 0.01, in which one of them is always *A*. Choosing *A* as a varying parameter can demonstrate the effect of increasing the power input on the complexity of the system. As can be seen in Figure 9, the complexity of the system is enhanced with the increase of *A*. In other words, boosting power input to the system can decrease its degree of regularity.

**Figure 10:**
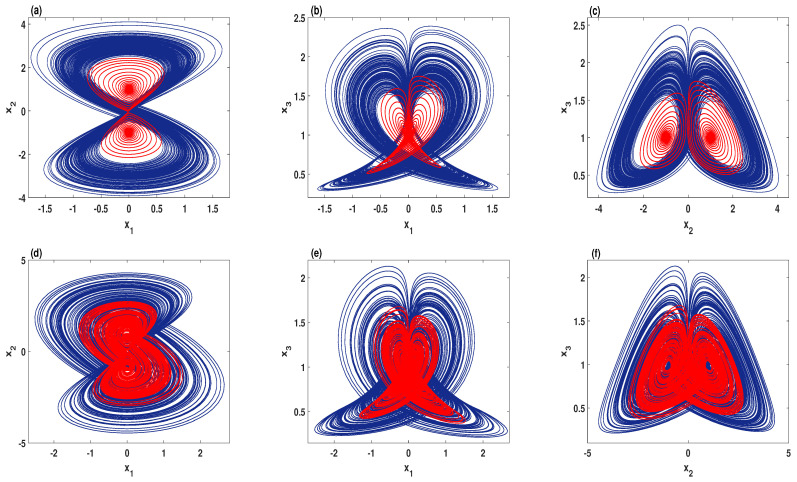
The phase portraits comparisons for A=0 (red), and A=0.105 (blue): (**a**–**c**) d=1, e=0.1, and h=2; (**d**–**f**) d=0.55, e=0.14, and h=2.

To perceive the effect of decreasing the degree of regularity on the dynamics of the system, Figure 10 plots the phase portraits comparisons with two values of the parameter *A*. In this figure, the trajectories with lower complexity (red color) are generated by the system (9) for A=0. In other words, these trajectories are the numerical solutions of the system (2), whereas the trajectories with higher complexity (blue color) are generated by system (9) for A=0.105. Thus, it can be observed that enhancing the complexity of the system (2) could change its nature from order to chaos or even expand its chaotic oscillations to occupy a wider region in the phase space, as shown in Figure 10a–f, respectively.

## 5. Performance Evaluations

This section evaluates the sensitivity and randomness of the chaotic sequences generated by the system (9) by cross-correlation coefficient and NIST-800-22 test.

### 5.1. Cross-Correlation Coefficient

As can be seen in Section 3, in the multistability regions, system (9) is sensitive to the initial conditions. However, it is important to examine the sensitivity performance of the system in the single stability regions, which are more desirable in cryptography applications [30]. Therefore, this subsection deals with the two cases that have appeared in Figure 10.

The sensitivity of system (2) and (9) can be estimated by the cross-correlation coefficient (CCF), which is given by

(16) $$\begin{array}{c} CCF\left(\alpha_{t},\beta_{t}\right)=\frac{\sum_{t=1}^{N}\left(\alpha_{t}-A(\alpha )\right)\left(\beta_{t}-A(\beta )\right)}{\sqrt{\sum_{t=1}^{N}{\left(\alpha_{t}-A(\alpha )\right)}^{2}\sum_{t=1}^{N}{\left(\beta -A(\beta )\right)}^{2}}}, \end{array}$$

where A(α) represents the mean value of the time series $\alpha_{t}$, meanwhile A(β) represents the mean value of the time series $\beta_{t}$. When the $CCF(\alpha_{t},\beta_{t})$ result is close to 0, then it can be indicated that these two time series are diverging.

**Figure 11:**
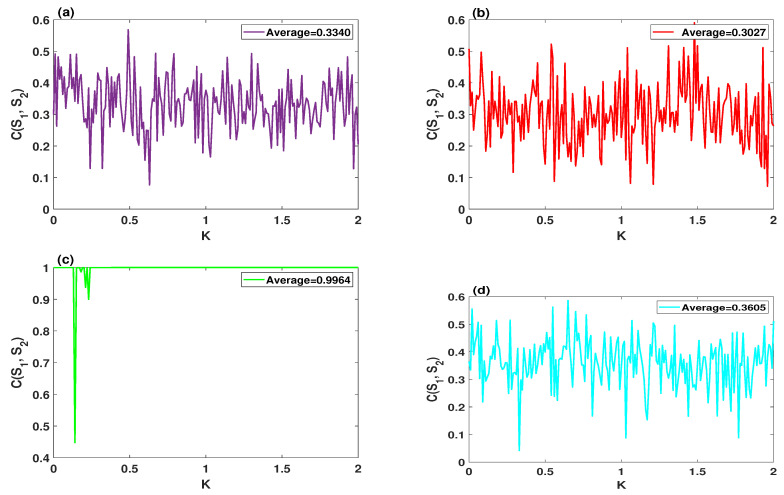
The cross-correlation coefficient (CCF) analysis of the systems (2) and (9) with (K+E,1,−1), where the step size of *K* is equal to 0.01: (**a**,**b**) the CCF of the systems (2) and (9) for d=0.5, e=0.1, h=2, A=0.105, respectively; (**c**,**d**) the CCF of the systems (2) and (9) for the parameters d=1, e=0.1, h=2, A=0.105, respectively.

When d=0.5, e=0.1, h=2, the sensitivity of systems (2) and (9) is illustrated in Figure 11a,b, respectively. In these two figures, the CCF is calculated between the original time series ($S_{1}$), and the modified one ($S_{2}$) which is created by contaminating one of the initial conditions of the system via a tiny error $E=5\times 10^{-5}$. Moreover, the sensitivity of the systems (2), and (9) are respectively depicted in Figure 11c,d, where the parameters are set as d=1, e=0.1, h=2, and A=0.105. The system (9) has a higher sensitivity to its initial conditions.

### 5.2. Chaos-Based Cryptographic Pseudo-Random Number Generator (PRNG)

A chaotic system with complex nonlinear behaviors is an appropriate source for producing PRNG. Generally, the randomness of the generated PRNG by chaotic systems is dependent on the characteristic properties of these systems such as the sensitivity of initial state, ergodicity, and unpredictability. As system (9) is extremely sensitive to its initial conditions and has high complexity performance, this system could be suitable for chaos-based PRNG.

**Figure 12:**
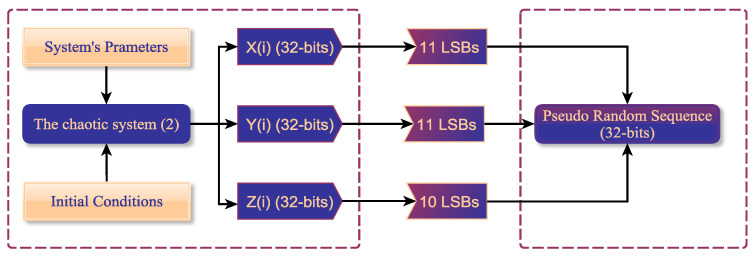
The flowchart of the proposed strategy for producing PRNG by the system (9).

To further examine the ability of the system (9) for generating valid PRNG, we propose a simple strategy, which directly uses the chaotic sequences as pseudorandom numbers. For the state variables of system (9), {X(i),Y(i),Z(i)|i=1,2,3…}, we convert each of their values to 32-bit binary stream using IEEE 754 float standard, as shown in Figure 12. Subsequently, the digital numbers in each binary stream are extracted from 22nd to 32nd (for X(i), and Y(i)), and from 23nd to 32nd (for Z(i)) to compose PRNG of 32-bits.

**Figure 13:**
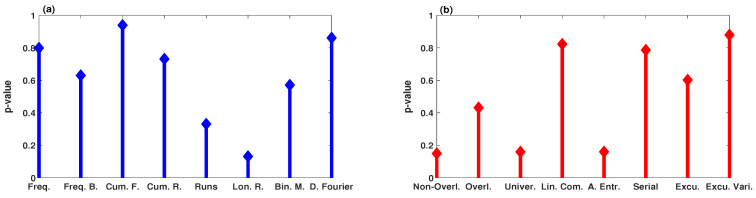
The *p*-values of the binary sequence generated by PRNG of the system (9): (**a**) Frequency, Block-Frequency, Cumulative Sums (Forward), Cumulative Sums (Reverse), Runs, Longest Run of Ones, Binary Matrix Rank, and Discrete Fourier Transform, respectively; (**b**) Non-overlapping Template, Overlapping Template, Universal Statistical, Linear Complexity, Approximate Entropy, Serial, Random Excursions, and Random Excursions Variant, respectively.

The validity of the generated PRNG by the system (9) can be checked using NIST-800-22 [41]. In NIST-800-22, there are 16 different statistical tests. However, a random sequence can pass the test if the corresponding *p*-value is greater than the experimental significance level of α. In our experiment, we generate a binary sequence of $10^{8}$ bits, which means that the obtained *p*-value should be greater than $\alpha^{-1}$ in each test. Figure 13 illustrates the NIST SP800-22 test results of PRNG that generates by the system (9). As can be seen, all the *p*-values are greater than $\alpha^{-1}$, which demonstrates the high randomness of the PRNGs using the system (9).

## 6. Conclusions

The dynamics of the 3D plasma perturbations model, which was proposed by Constantinescu et al. to describe the pellet injection, was investigated by time series, Lyapunov exponents, bifurcation diagrams, and basins of attraction. Besides that, the stability of its equilibria has been analyzed. The mathematical analysis has shown that the system has three equilibria in which there is always an unstable equilibrium point, which indicates that the attractors type of this system is self-excited. To discover the chaotic regions that show no transient chaos, the two-dimensional MLE has been applied. Furthermore, we have conducted comprehensive research on the dynamics, complexity, and randomness of the system after boosting power input. Simulation results have demonstrated that the system could produce multiple coexisting attractors, high complexity, and randomness.

## Figures and Tables

**Table 1:** The Lyapunov exponents (LE) and Kaplan–Yorke dimension (DKY) of the attractors shown in Figure 6.

Figures	Initial Conditions	LE	DKY
Figure 6a,b	(1,1,−1) (blue)	(0.0258,0,−1.1248)	2.0229
	(2,1,−1) (red)	(0.0270,0,−1.1285)	2.0239
Figure 6c,d	(1,1,−1) (blue)	(0,−0.0176,−1.1158)	1.9842
	(2,1,−1) (red)	(0,−0.0177,−1.1161)	1.9841

## Data Availability

Not applicable.
